# BIDCHIPS: bias decomposition and removal from ChIP-seq data clarifies true binding signal and its functional correlates

**DOI:** 10.1186/s13072-015-0028-2

**Published:** 2015-09-17

**Authors:** Parameswaran Ramachandran, Gareth A. Palidwor, Theodore J. Perkins

**Affiliations:** Regenerative Medicine Program, Ottawa Hospital Research Institute, K1H 8L6 Ottawa, Canada; Department of Biochemistry, Microbiology and Immunology, Faculty of Medicine, University of Ottawa, K1H 8M5 Ottawa, Canada

## Abstract

**Background:**

Unraveling transcriptional regulatory networks is a central problem in molecular biology and, in this quest, chromatin immunoprecipitation and sequencing (ChIP-seq) technology has given us the unprecedented ability to identify sites of protein-DNA binding and histone modification genome wide. However, multiple systemic and procedural biases hinder harnessing the full potential of this technology. Previous studies have addressed this problem, but a thorough characterization of different, interacting biases on ChIP-seq signals is still lacking.

**Results:**

Here, we present a novel framework where the genome-wide ChIP-seq signal is viewed as being quantifiably influenced by different, measurable sources of bias, which can then be computationally subtracted away. We use a compendium of 123 human ENCODE ChIP-seq datasets to build regression models that tell us how much of a ChIP-seq signal can be attributed to mappability, GC-content, chromatin accessibility, and factors represented in input DNA and IgG controls. When we use the model to separate out these non-binding influences from the ChIP-seq signal, we obtain a purified signal that associates better to TF-DNA-binding motifs than do other measures of peak significance. We also carry out a multiscale analysis that reveals how ChIP-seq signal biases differ across different scales. Finally, we investigate previously reported associations between gene expression and ChIP-seq signals at transcription start sites. We show that our model can be used to discriminate ChIP-seq signals that are truly related to gene expression from those that are merely correlated by virtue of bias—in particular, chromatin accessibility bias, which shows up in ChIP-seq signals and also relates to gene expression.

**Conclusions:**

Our study provides new insights into the behavior of ChIP-seq signal biases and proposes a novel mitigation framework that improves results compared to existing techniques. With ChIP-seq now being the central technology for studying transcriptional regulation, it is most crucial to accurately characterize, quantify, and adjust for the genome-wide effects of biases affecting ChIP-seq. Our study also emphasizes that properly accounting for confounders in ChIP-seq data is of paramount importance for obtaining biologically accurate insights into the workings of the complex regulatory mechanisms in living organisms. R and MATLAB packages implementing the framework can be obtained from http://www.perkinslab.ca/Software.html.

**Electronic supplementary material:**

The online version of this article (doi:10.1186/s13072-015-0028-2) contains supplementary material, which is available to authorized users.

## Background

Transcriptional regulation has been the focus of intense investigation ever since seminal works on gene regulatory mechanisms appeared in the literature about half a century ago [1, 2]. The past few decades have seen steady progress, revealing important insights that not only identify the main players of transcriptional regulation, such as transcription factors (TFs) [3], core promoters [4], enhancers [5], and silencers [6], but also begin to unravel the complex interplay existing between these entities [7]. On the one hand, these studies substantially enhance our understanding of fundamental biological processes, such as differentiation and development [8], while, on the other hand, they shed light on the deviations from normal expression patterns (attributable to misregulation) that are often responsible for initiating disease states such as cancer [9–11].

Among the deluge of high-throughput quantitative technologies that have facilitated transcriptional regulation studies in recent years, a powerful and arguably the most popular platform today is ChIP-seq (Chromatin ImmunoPrecipitation followed by massively parallel sequencing) [12]. This is a high-throughput method for the genome-wide in vivo identification of the binding sites of DNA-associated proteins. In the ChIP step, crosslinked DNA–protein extracts are sheared and enriched by antibodies specific to the protein of interest. The purified DNA fragments are then identified through high-throughput sequencing and mapped back to the organism’s canonical genome for further computational analysis [13, 14].

While the ChIP-seq technology has advantages compared to its microarray-based counterpart (ChIP-chip), such as very high resolution and sensitivity, it also poses significant challenges stemming from both inherent biases in the design of the technology [15] as well as differences in how individual experiments are conducted [16]. For example, GC-rich fragments are often overrepresented in ChIP-seq datasets, giving rise to the GC-content bias [17, 18]. There is also a mappability bias that occurs due to differences in sequence complexity [19]. Since only uniquely mappable reads are typically retained in ChIP-seq datasets, reads falling in low complexity regions are predisposed to being discarded, resulting in the bias. Furthermore, differences in chromatin structure can lead to a chromatin accessibility bias. For instance, heterochromatin is more tightly packed compared to euchromatin, resulting in accessibility differences and decreased read density [20, 21]. Other sources of biases, such as PCR amplification and nucleic acid isolation, also contribute to distortions in the ChIP-seq signal [19, 22, 23]. It is thus important to address the effects of these biases because they can influence both the identification and the prioritization of putative transcription factor binding sites or regions of chromatin modification.

Many peak-calling algorithms—methods for automatic detection of enriched genomic regions in a ChIP-seq experiment—account for these biases using either a parallel input DNA (iDNA) control that has not been subjected to the immunoprecipitation step, or a mock ChIP sample where a non-specific IgG antibody is used [13, 24–27]. In both cases, the assumption is that the ChIP and the control samples have the same biases which, if untrue, would render the methods ineffective. Other algorithms address specific biases in a somewhat heuristic manner by either modeling and removing them or introducing adjustments to local read positions and/or counts in the overall ChIP-seq signal profile [18, 19, 28].

**Fig. 1:**
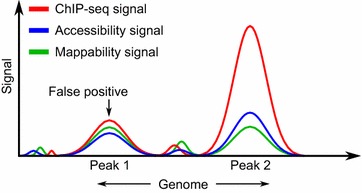
A signal-processing view of ChIP-seq data. Here, we show two ChIP-seq peaks (potential regions of enrichment) with different signal levels. Let us assume that after accounting for mappability and accessibility, what is left in the overall signal corresponds to true binding. Peak 1 is unlikely to be a genuine peak because only a small portion of the overall signal may correspond to binding. Peak 2, on the other hand, is much more likely to indicate true binding, as a relatively smaller share of the overall signal is attributable to mappability and chromatin accessibility, leaving a much larger portion for true binding

Although such methodologies have been partly successful in correcting for various biases, it is not always clear which biases they account for and which they do not. To provide clarity, a large-scale genome-wide study quantifying the composition of the ChIP-seq signal in terms of the biases and other factors such as notions of control would be very useful, and is missing from the current literature. Such a study would help answer two questions. One, is it reasonable to view ChIP-seq data as a composite genome-wide signal, capable of being decomposed (or separated) into its constituent parts—with each part attributable to binding, biases, or other non-binding factors such as controls? And, two, if such a decomposition is possible, then how much of the signal can be attributed to each factor? In other words, can we quantify the roles played by the constituent factors in making up the ChIP-seq signal? Figure 1 shows a schematic of a ChIP-seq signal in peak regions where the biases due to mappability and chromatin accessibility are represented as component signals. Such a signal-processing view of ChIP-seq data would help identify and separate the binding and non-binding portions of the signal, ultimately leading to more accurate biological interpretation of the data.

In this study, we present a novel framework that allows the quantification, both individually and jointly, of various biases that act on genome-wide ChIP-seq signals. We set out to look at ChIP-seq data as a signal with quantifiable components, also referred to as *predictors* in the ensuing text. We first construct a staged regression model using a large set of human ENCODE [29] ChIP-seq datasets (123 in total) from the three Tier 1 cell lines to quantify the ChIP-seq signal in terms of five predictors: Mappability, GC-content, chromatin accessibility, input DNA control, and IgG control. The quantification reveals that a large part of the overall ChIP-seq signal is attributable to these background factors. We then carry out a multiscale analysis using the model to quantify the trends of ChIP-seq signal composition across different scales, which also highlights the complex relationships existing between the predictors. After establishing a background model in this manner, we then proceed to use the model to separate the binding and non-binding portions of the ChIP-seq signal. We show that the resulting corrected binding signal is able to rank peaks better than peak-callers such as MACS. Finally, we use our model to investigate previously reported associations between gene expression and TF and histone mark (HM) ChIP-seq signals in small windows centered at TSSs [30, 31]. Other reports [32–34] have questioned the validity of such associations, recommending caution while interpreting ChIP-seq signals in highly expressed genomic regions. Motivated by these studies, we predict expression using our model with various predictor sets such as standalone TFs, standalone HMs, and TFs and HMs combined with controls. Our results not only confirm the concerns raised in the cautionary studies mentioned above, but go a step further in providing concrete evidence that many of the associations between TF signal around TSSs and gene expression can really be attributed to confounding factors, particularly chromatin accessibility. Furthermore, we show that our model can account for these confounding factors, thereby clearly differentiating ChIP-seq signals that truly associate with gene expression from those that do not.

## Results

### A large portion of the ChIP-seq signal does not correspond to true binding

To test the hypothesis that ChIP-seq signals contain components not related to true transcription factor binding or chromatin marks, we turned to the wealth of human ENCODE data [29]. The ENCODE consortium has studied transcription factor binding and chromatin state in a large number of cell lines, but three “Tier-1” cell lines were studied especially thoroughly in human: GM12878, a lymphoblastoid cell line, H1-hESC, human embryonic stem cells, and K562, an immortalized myelogenous leukemic cell line. Of the available ChIP-seq assays, we identified 30 TFs and 11 HMs that were assayed in all three cell lines. We decided to use those 123 datasets as the basis for our investigation. We also selected a set of five other genomic signals that we thought might influence, or be present within, the ChIP-seq data: mappability, GC-content, chromatin accessibility, and input DNA and IgG control signals. Because the majority of the mapped reads we processed were 36 base pairs long, we obtained a 36-base-pair mappability track from the UCSC Table Browser [35] and binarized it, so that each base pair in the genome is designated simply as either mappable or unmappable. Similarly, our binarized GC-content signal reports whether each base pair in the genome is G/C (1) or A/T (0). Our mappability and GC-content signals are derived from the reference human genome (hg19), and we treat them as being identical for all three cell lines. This is an approximation to the truth for a number of reasons. First, any genome assembly has errors in it, arising because of a variety of technical reasons [36]. Second, the three cell lines come from different human individuals, each with their own genetic variations. Third, the K562 cell line in particular, being cancer-derived, has numerous mutations that are not reflected in the hg19 assembly. In fact, even at the karyotypic level, this cell line is wildly abnormal, containing three copies of many chromosomes along with complex translocations involving multiple chromosomes [37]. Despite these known differences, no specialized genome assemblies for the three Tier-1 cell lines are presently available, and so we rely on hg19 as the best available proxy. In contrast, we treat chromatin accessibility as being different across cell lines and, accordingly, chose to represent it based on the cell line-specific DNaseI hypersensitivity high-throughput sequencing datasets generated by ENCODE. In the same manner, we obtained from ENCODE the cell line-specific input DNA and IgG control read sets that are commonly used for peak-calling. The genome-wide signals for DNaseI hypersensitivity, input DNA, and IgG control depend on mapping short reads to the hg19 human genome assembly which, as we have noted above, does not entirely match the cell lines we study. Still, these signals are cell line-specific, and therefore should reasonably reflect differences in chromatin accessibility and other properties between the cell lines that can affect ChIP-seq signals.

For our first analysis, we divided the genome into consecutive windows of (2^7^ + 1) = 129 base pairs. This is smaller than the majority of TF peaks and histone mark-enriched regions, so it should capture any meaningful variations in these signals, while still allowing for some regional averaging to reduce noise. We tabulated read counts for all the ChIP-seq, DNaseI, input DNA, and IgG control datasets in these windows, as well as mappable bases and GC-content. Our goal was to determine how much of the ChIP-seq signals can be explained in terms of the other five factors, which we will call *predictors*. However, the predictors themselves are partially correlated. For example, unmappable regions cannot have DNaseI, iDNA, or IgG reads mapped to them, so the latter signals will also be zero in those regions. To get around this problem, we conducted a staged regression analysis. We first built a linear model predicting ChIP-seq reads from mappability alone, and determined how much of the ChIP-seq signal was explained. Then, we added GC-content to the model, then DNaseI, and so on. With each added feature, we track how much additional predictive power we gain, and only that gain is attributed to the added feature. In this way, for example, we attribute to the DNase I hypersensitivity predictor only what it explains about the ChIP-seq data that is not already explained by mappability or GC-content. We chose to prioritize mappability first because of its well-known influence on read densities and because it is a cell state-independent feature (modulo our comments above). Similarly, GC-content is cell state-independent, so we chose it next. Chromatin accessibility was prioritized next, due to its known connections to transcription factor binding [38]. Of the two common peak-calling controls, we expected input DNA to be less specific than IgG, so we wanted to account for its influence first. IgG control, which is procedurally most similar to performing a TF ChIP-seq, therefore, came last in the order of priority.

**Fig. 2:**
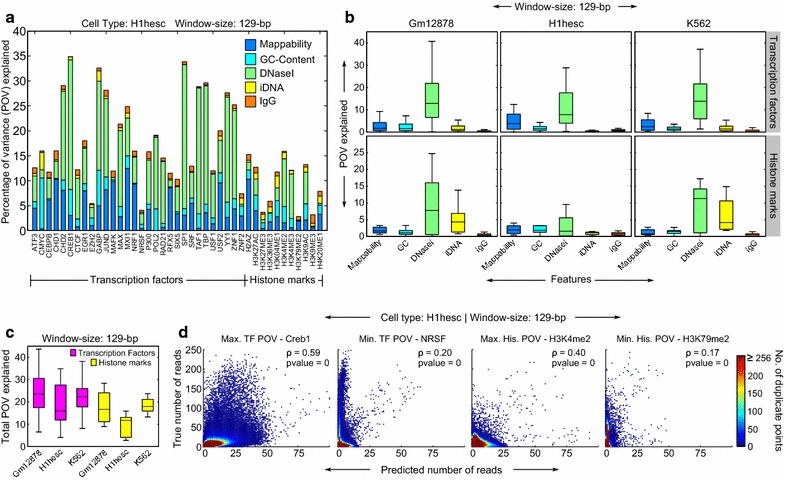
Power of different predictors for predicting the strength of the ChIP-seq signal. All plots correspond to a window size of 129 bp. **a** *Stacked bar* plot for the H1-hESC cell line showing the predictive power of different predictors in terms of the POV explained. **b** *Box plots* showing the variability in the predictive power of the predictors across datasets in a given cell line. *Boxes* represent the interquartile range, which measures the spread of the data. The *top* whisker ends at q_3_ + w(q_3_ − q_1_) and the *bottom* whisker ends at q_1_ − w(q_3_ − q_1_), where w = 1.5 and q_1_ and q_3_ are the 25th and the 75th percentiles, respectively. **c** *Box plot* showing the variability of the total predictive power of all the predictors combined. Data are grouped such that we have one *box* per combination of cell line and ChIP-seq type (TF or histone marks). **d** *Scatter plots* showing the correlations between true and predicted number of reads in individual windows for chosen cases: maximum and minimum total POV values for each of the TF and HM groups in **a**, all from the H1-hESC cell line. The name of the TF or HM is also indicated. For plotting purposes, the predictions were rounded to the nearest integer for consistency with the true read numbers (which are always integers). The plots have also been color coded to indicate duplicate points. This reveals large clusters at the lower left corners of the plots indicating many windows having very few to no reads. The Pearson’s rho (ρ) along with the *p*-values are also shown

Figure 2a shows stacked bar plots of the percentages of variance explained (POVs) for all the ChIP-seq datasets corresponding to the H1-hESC cell line, for regression models successively adding more features. For most of the datasets (34 out of 41), mappability and chromatin accessibility together share most of the total predictive power held by all the predictors combined. For example, for ATF3, the five predictors combined explain 12.61 % of the total variance in the dataset. Of this, mappability explains 4.49 % and chromatin accessibility (measured using DNaseI hypersensitivity) explains an additional 5.11 %, amounting to a total of 9.6 %. In contrast, the two notions of control, IgG and input DNA, explain significantly smaller percentages of variance (0.53 and 1.13 %, respectively), after mappability and chromatin accessibility have been accounted for. The total variance explained, as well as the portions explained by individual predictors, varies substantially between different datasets. For instance, only 4.09 % of the NRSF signal is explained by all predictors combined, with only 0.46 % coming from mappability and 2.14 % from chromatin accessibility. By contrast, 34.87 % of the CREB1 signal is explained by the predictors, with 3.04 % attributable to mappability and 25.97 % to chromatin accessibility. Similar trends hold for the other two cell lines (Additional file 1: Figures S1, S2). Together, these results show that a large portion of the ChIP-seq signal comes neither from specific aspects of the control data nor from the binding itself, but rather from other factors such as mappability and chromatin accessibility. Particularly interesting is the consistently large predictive power of chromatin accessibility. Equally interesting, and in fact surprising, is the significantly small predictive power wielded by the controls IgG and input DNA. Thus, our first conclusion is that mappability and chromatin accessibility are the two strongest determinants of the strength of the ChIP-seq signal in a given genomic region, at least at the relatively small scale of 129-bp windows. Although numerous previous works have considered mappability as a factor influencing read densities [15, 19, 22], and chromatin accessibility is known to influence transcription factor binding [20], our analysis provides systematic confirmation and quantification of these influences using a large compendium of ENCODE datasets. Complete lists of POV values for all datasets and cell lines are presented in Additional file 2: Table S1, Additional file 3: Table S2, and Additional file 4: Table S3.

Figure 2b summarizes the predictive power of individual predictors across datasets, separated by cell line and by transcription factor versus chromatin mark. In all cases, DNaseI hypersensitivity shows the largest spread among the predictors. It also has the largest median predictive power in all cases except H1-hESC histone marks, where mappability and GC-content have higher median predictive power. This means that although chromatin accessibility is generally the largest influence on these ChIP-seq signals, its importance varies widely across datasets. For the transcription factor ChIP-seq signals, mappability is the second most important factor, as mentioned above. For the histone mark ChIP-seq signals in the Gm12878 and K562 cell lines, however, the input DNA signal is generally the second most important after chromatin accessibility, and more important therefore than mappability.

The total predictive power from all predictors (the stack heights in Fig. 2a) is summarized in Fig. 2c. Each box in Fig. 2c represents either TFs or histone marks for a particular cell line. The total POV values across all datasets and cell lines range from 2.82 to 43.62. Overall, the predictability of TF data is higher than the predictability of histone data (Wilcoxon rank-sum test, *p*-value 0.0002). Across cell lines, both the Gm12878 and the K562 predictabilities are better than the predictability in H1-hESC (Wilcoxon signed-rank test, *p*-values 0.00002 and 0.0004, respectively), while they themselves are not significantly different from each other (*p*-value 0.41). Thus, the total percentage of variance explained varies by cell line as well as between transcription factors and histone marks.

Figure 2d shows scatter plots of the true and the predicted numbers of windowed read counts for selected cases. We chose the TF and the HM corresponding to the maximum and the minimum total POV value for plotting. Trends are as expected, with the TF cases showing stronger correlations than HM cases.

### Predictive power and predictability improve at larger scales

**Fig. 3:**
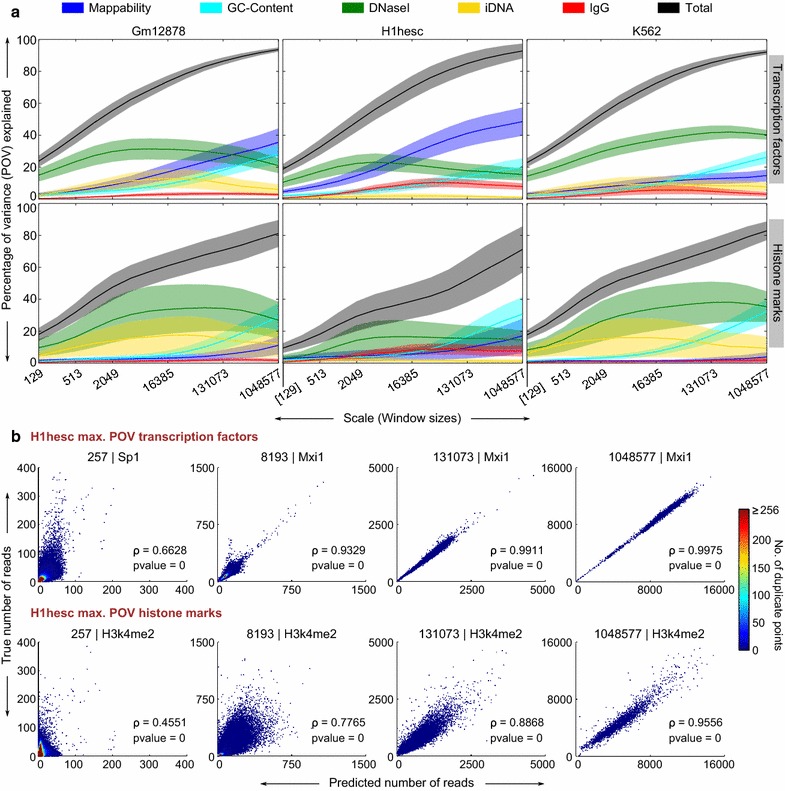
Multiscale analysis of the predictive power of predictors and the overall predictability of ChIP-seq data. **a** Patch plots showing the variation of predictor power and predictability across multiple scales. For each patch, the *dark central line* represents the mean across datasets and the surrounding *lighter shade* represents two standard deviations on either side of the mean. **b** Scatter plots of the predicted and true read numbers in windows for selected TFs and HMs across multiple scales. For each scale shown, the specific TF and HM are those with the maximum total POV value in the respective group. Similar to the scatter plots in Fig. 2, the predictions have been rounded for consistency with the true read numbers and the points are color coded to indicate duplicate points. The Pearson’s rho (ρ) along with the *p*-values is also shown

The analysis of the previous section, employing 129-bp windows, illustrates variability in ChIP-seq signals on a small scale. However, for some transcription factors and regulatory complexes, average peak size can be many times larger [39, 40]. Further, signals for chromatin marks are usually diffuse, spanning from several nucleosomes to large domains covering multiple genes [41, 42]. Thus, it is important to consider influences on ChIP-seq signals at a variety of scales larger than 129 bps. In this section, we observe a remarkably consistent trend wherein the predictive power of some predictors, and consequently the total predictability of the ChIP-seq data, improves significantly as the scale becomes larger. We applied the regression model described above across all datasets and over a range of window sizes in increasing powers of 2, from (2^7^ + 1) to (2^20^ + 1) ≈ 10^6^ bp. Figure 3a summarizes the results of this multiscale analysis, where each subpanel corresponds to either transcription factors or histone marks for a particular cell line. The curves in the subpanels represent the trends of the total predictive power (black) and the predictive power of the five predictors (colors) obtained using staged regression across the range of scales. The dark central line of each patch denotes the mean across the datasets in the group, while the surrounding lighter color represents two standard deviations on either side of the mean. For example, in Gm12878 cells, the total POV across TFs went from 23.82 ± 3.49 at (2^7^ + 1) bps to 93.64 ± 1.25 at (2^20^ + 1) bps. The POV specifically by mappability went from 3.00 ± 1.05 to 35.6 ± 8.6 over the same range of window sizes.

The first striking observation is that the overall predictability of ChIP-seq data (in terms of the total POV explained) increases dramatically with increasing scale, for all three cell lines and both data groups. At the largest scale of analysis, which uses windows of just over a million base pairs, approximately 90 % of the variability in TF ChIP-seq data and 70–80 % of the variability in HM data is explained, mostly by chromatin accessibility, mappability, and GC-content. The predictive power of mappability is high and increasing with scale for the TFs in the Gm12878 and H1-hESC cell lines. In the other four cases, GC-content is more important than mappability and increases more strongly with scale. The predictive power of chromatin accessibility (DNaseI hypersensitivity) increases at smaller scales, but then flattens out or even decreases at the larger scales. This profile is also shared by the predictive power of the input DNA and IgG features, although the total amount of variance they explain is smaller. Thus, as in our 129-bp analysis, the two standard ChIP-seq peak-calling controls, input DNA and IgG, have relatively little predictive value once the other factors are taken into account.

Between the two data groups, histone marks have larger spreads around the mean curve than the TFs in general, meaning the predictability of the histone mark signals varies more widely. The iDNA predictor has a distinctly thinner spread for H1-hESC compared to the other two cell lines, whereas the total POV explained shows the opposite effect—spread for H1-hESC is thicker than those for the other two cell lines.

Figure 3b shows scatter plots of the true and the predicted numbers of windowed read counts at four different scales. For each scale, we chose to display the plot corresponding to the TF or HM with the maximum total POV value. This corresponded to the TF Mxi1 (except for the lowest scale where it was Sp1), and the histone mark H3k4me2. In agreement with Panel A, for both TFs and HMs, the predictions become more accurate as the scale becomes larger.

### Removing non-binding influences produces a better estimate of true binding signal

The results thus far demonstrate that multiple non-binding influences on ChIP-seq signals can be identified and quantified. Intuitively, if we subtract away the non-binding components of the ChIP-seq signal using our genome-wide model, what remains should correspond more closely to the true binding signal. Of course, we do not know the true strength of TF binding across the genome. However, previous studies suggest that higher affinity TF peaks are more likely to harbor DNA-binding motifs or even multiple such motifs [43, 44]. Thus, we decided to look for correlations between estimated binding signal strength based on our regression modeling and TF-DNA-binding motif frequencies. Of the 30 TFs in our dataset, we identified 17 for which a clear DNA-binding motif was available in the JASPAR database [45] (see Additional file 5: Table S4 for TF and motif list). We obtained peaks for those TFs by running MACSv1.4.2 (the latest stable release at the time of our analysis) with default parameters. Since the peaks are usually asymmetric with respect to their summits and can differ in width significantly, for the sake of consistent analysis across datasets, we redefined a peak as a symmetric window around the summit location. Specifically, we took windows of (summit ±64) base pairs as the peaks. This resulted in 129-bp peaks, which was consistent with the scale of our initial ChIP-seq analysis and safely within the average peak size across the different datasets. We estimated true binding signal in peaks by subtracting the predicted read numbers based on our genome-wide background model from the corresponding true read numbers. Intuitively, this difference corresponds to the portion of the ChIP-seq signal not explained by other, potentially confounding factors, and thus is more likely to represent true binding. We refer to this as the estimated binding signal (B). Then, we used FIMO [46] with default parameters along with in-house scripts to identify and count motif occurrences in the peaks—we call this signal M (see “Methods” for complete details of the FIMO runs). Finally, we computed the Pearson’s correlation coefficient between signals B and M. As a basis for comparison, we also correlated signal M with two other measures of the binding signal: (1) the raw read numbers, signal R, and (2) the MACS peak scores, signal S (these are scaled log *p*-values). Intuitively, a stronger true binding signal should be associated with a higher motif occurrence; so the estimate of the binding signal (B, R, or S) that best correlates with motif frequencies (M) can be taken as the best estimator of true binding.

**Fig. 4:**
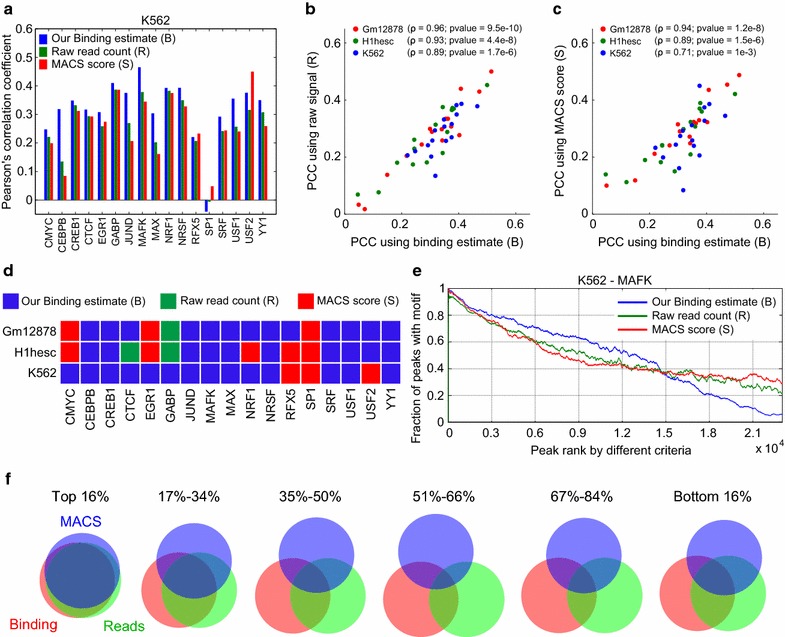
Correlation analysis with motif counts in peaks and peak-ranking analysis. **a** Pearson’s correlation coefficients between different model scores in peaks and DNA-binding motif counts computed for all 17 TFs from the K562 cell line. **b**, **c** Scatter plots of the PCC values for estimate B with the PCC values for the other two estimates. **d** Grid plot identifying the binding estimate that correlates best with motif frequencies for a given cell line and transcription factor. **e** Curves showing the fractions of peaks within a moving window of 1000 peaks that contain at least a single motif, when ranked using different criteria. **f** Venn diagrams showing the degrees of overlap between corresponding sextiles of peaks ranked by different criteria

The results for the K562 cell line are shown in Fig. 4a (see Additional file 1: Figures S3, S4 for results corresponding to the other cell lines). Cases B, R, and S are indicated by different colors as shown. We see that the correlations of the different binding estimates to motif counts vary widely for the different transcription factors, but that the three estimates largely vary in concert. For example, all three are poor for CMYC in GM12878 and H1-hESC, whereas all three are relatively good for CREB1. This trend is particularly apparent in Figs. 4b, c, where we show scatter plots of the Pearson’s correlation coefficient (PCC) values for estimate B with the PCC values for the other two estimates. For the majority of datasets, all three estimators have correlations between 0.2 and 0.4, which are substantial and statistically significant (*p*-values ranging from 0 to 1.77 × 10^−48^). Across the cell lines, the three estimates co-vary best for Gm12878, followed by H1-hESC and K562 in that order. Figure 4d shows a ternary grid, where the color of a square identifies the estimate that correlates best with motif frequencies for a given cell line and transcription factor. Signal B outperforms the other two in 37 out of a total of 51 cases, which is a statistically significant fraction (*p* = 1.2 × 10^−8^ by ratio test, under the null hypothesis of no difference between the methods). Of the remaining cases, the split between methods R and S is 3 to 11. Very similar results are obtained if we correlate B, R, and S to the presence (count > 0) or absence (count = 0) of motifs in the peak (Additional file 6: Table S5). Correlations involving actual motif counts are a bit better for the majority of cases (about two-thirds) compared to correlations involving just motif presence or absence.

As a further evaluation of the performance of the three estimates, we ranked the peaks in order of best to worst scores obtained using the three estimates. Then, we computed the fraction of peaks within a moving window of 1000 peaks that contained at least one motif (the notion of presence/absence of motifs is more natural for this scenario). The resulting trends for the three estimates corresponding to an example TF MAFK from the K562 cell line are plotted in Fig. 4e. A good ranking of the peaks is one in which peaks with a DNA-binding motif are near the top, and peaks lacking motifs are near the bottom. Accordingly, in Fig. 4e, we see that the curve corresponding to our estimate B is above the other curves in the left half of the plot and below the other curves in the right half, indicating a better ranking than the other two estimates. In other words, our binding estimate B concentrates motif-rich peaks more towards the top and motif-poor peaks more towards the bottom of the list, leading to a better overall grouping of the peaks.

Finally, the Venn diagrams in Fig. 4f picture the degree of overlap between the top 1/6th of peaks, the next 1/6th of peaks, and so on, as ranked by the three different criteria. All three methods agree pretty well on what are the top peaks. But then as one goes down the list disagreement grows, until one gets to the end where the methods start agreeing again on what are the worst peaks.

Thus, overall, in a statistically significant manner, our model is able to more effectively separate out the non-binding components of the ChIP-seq signal compared to the other competing models considered. The motif analysis presented in this section amounts to a biological validation of our model, thereby demonstrating its utility in the accurate identification of TF-binding sites.

### Chromatin accessibility explains most of the association between specific transcription factors and gene expression, but general factors have genuine predictive power

A number of studies have investigated the predictability of gene expression using factors such as TF/histone binding activity in peak regions and chromatin accessibility [47, 48]. Other studies have forgone peak-calling, and simply used raw TF ChIP-seq signals (read counts) in the vicinity of transcription state sites (TSSs) to predict gene expression [30, 31]. In this latter camp, some startlingly high, and statistically significant correlations have been found between the ChIP-seq signals of many individual TFs and gene expression. One possible explanation for these observed associations is that quantitative TF-binding levels at the TSSs directly regulate gene expression. However, there are several reasons to doubt this explanation, even if the correlations themselves are not in doubt. First, the signals of many different TFs are found to be associated to expression, even when those TFs themselves are often not known to bind in any coherent or coordinated fashion. Second, most genuine TF-binding activities are believed to occur in peak regions. Therefore, associations that do not explicitly involve peaks, but rather look only at the signal around the TSSs irrespective of whether a peak is present or not, may not have anything to do with true binding. Third, gene expression levels are known to directly relate to chromatin accessibility [49, 50] with a large proportion of TSSs being in open chromatin regions [48]. This fact, combined with our above result that ChIP-seq signals contain a substantial component reflecting chromatin accessibility, alludes to chromatin accessibility acting as a confounder in these associations. Due to all these reasons, it is compelling to consider the alternative possibility that the observed association between gene expression and TF ChIP-seq signal in a small region centered at the TSSs may actually be explained by relationships between expression and background/non-binding signal in the ChIP-seq data.

To systematically investigate the cause of these associations, we first predicted expression for the GENCODE [51] v19 genes (annotation data downloaded from the UCSC Table Browser) in the three cell lines using each TF and HM dataset individually. Specifically, we chose the numbers of CAGE (Cap Analysis Gene Expression) [52] reads in windows of size 129 bps (TSS ±64 bps) to represent gene expression. In the same windows, we counted ChIP-seq reads for the TF and HM datasets. We constructed linear regression models predicting gene expression based on each TF’s and HM’s read counts. A tenfold cross-validation analysis was carried out for each predictor to estimate prediction accuracy and establish confidence intervals for those estimates.

**Fig. 5:**
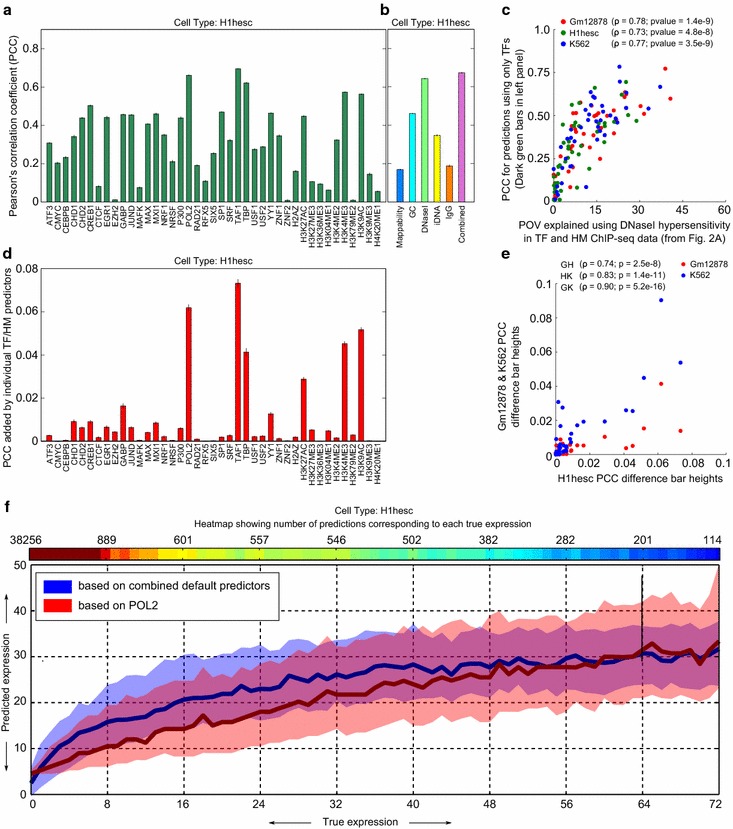
Predictability of gene expression using TFs, HMs, and core predictors. **a**, **b** Pearson’s correlation coefficient between true and predicted gene expression values for different predictor combinations. *Left panel*: PCCs for individual TFs and HMs. *Right panel*: PCCs for the individual core predictors as well as for the case when all core predictors are combined. **c** Scatter plots of the predictive power of chromatin accessibility in TF and HM ChIP-seq data (from Fig. 2a) and the predictability of gene expression using individual TFs and HMs (**a**). **d** Additional predictive power in terms of increase in PCCs caused by the combination of individual TF/HM predictors with core predictors. **e** Scatter plot showing PCC increases in the Gm12878 and K562 cell lines (y-axis) versus those seen in H1-hESCs (x-axis). **f** Patch plots showing the prediction accuracy of gene expression for two chosen cases of predictors: combined core predictors and POL2. X-axis represents true expressions in terms of CAGE reads and the y-axis represents predicted expressions using different predictor sets as indicated. The median (*central* *line*) and the interquartile range (*shaded background*) are shown for each case. The heat map on top shows the point densities, i.e., the number of windows with a given true expression value. This distribution is heavily weighted towards the lower end, due to most genes being expressed at low to moderate levels. For all the plots, gene expression is measured in terms of the number of CAGE reads in 129-bp windows around TSSs. For all predictions, a tenfold cross-validation was carried out

Figure 5a shows the accuracy of the predictors in H1-hESCs, in terms of the Pearson correlation coefficient (PCC) between predicted and actual gene expression. The different TFs and HMs have widely varying power to predict gene expression. Nevertheless, many have statistically significant predictive power, with correlations between predictions and actual gene expression ranging between 0.2 and 0.5 for many TFs. (Small black marks at the top of each bar give the 95 % confidence intervals based on cross-validation.) Several general TFs, including Pol2, TAF1, and TBP have correlations over 0.6, and two HMs, namely, H3K4me3 and H3K9ac, are not much behind. These results confirm previous findings of correlations between gene expression and numerous TF and HM ChIP-seq signals at TSSs. These trends are mostly consistent across different cell lines (see Additional file 1: Figures S5, S6).

To understand these results more deeply, in Fig. 5b (and in Additional file 1: Figure S7), we present the results of similarly predicting gene expression based on mappability, GC-content, DNaseI hypersensitivity reads, iDNA reads, and IgG reads (which we now collectively call our *core predictors* to distinguish them from TF or HM signals used as predictors). As with TFs and HMs, the power of different core predictors to predict gene expression using only their signals at the TSSs varies significantly, with mappability and IgG control being at the low end, input DNA and GC-content being midrange, and chromatin accessibility having the highest predictive power. Surprisingly, even when the core predictors are used together, the combined predictive power does not improve much compared to using chromatin accessibility alone. This strongly suggests that, in small windows around the TSSs, the chromatin state effect dominates over all the other predictors. Indeed, only two general transcription factors (in H1-hESC) predict expression better than the chromatin accessibility signal (Pol2, *p* = 0.003, and Taf1, *p* = 0.0002, by Wilcoxon rank-sum test). Conversely, no TSS ChIP-seq signal of a “specific” TF or HM on its own displayed a higher correlation to gene expression than the chromatin accessibility signal. Indeed, all were substantially worse predictors, with *p*-values of 0.0002 or smaller.

As a further test of our conjecture that the chromatin accessibility component of TSS ChIP-seq signals is responsible for their correlation to gene expression, we present the scatter plot in Fig. 5c. The plot shows, for the three cell lines, the correlation between the power of chromatin accessibility to predict genome-wide TF and HM ChIP-seq data (the light green components from Fig. 2a) and the PCC values for expression predictions using the TF and HM signals one at a time (the dark green components from Fig. 5a). We see that when different TF or HM ChIP-seq signals have a greater chromatin accessibility component, their TSS ChIP-seq signals also tend to have a greater association to gene expression.

A stronger and more quantitative evidence of a meaningful relationship between gene expression and a TF/HM ChIP-seq signal at TSSs would be the additional predictive power that ensues when an individual TF/HM predictor is combined with our core predictors. Figure 5d shows this measure for H1-hESC in terms of the increase in correlation coefficient obtained by adding individual TFs and HMs one at a time to the combined core predictors. This plot clearly shows that hardly any TF or HM signals are substantially related to gene expression. Some improvements are statistically significant due to the large number of genes involved, but the increases in correlation coefficient are less than 0.02 in nearly all cases. Only the general factors Pol2, TAF1, and TBP, and the histone marks H3K27ac, H3K4me3, and H3K9ac stand out as adding substantial information beyond our core predictors. Similar results are obtained in the Gm12878 and K562 cell lines, which are shown as a scatter plot in Fig. 5e. Here, we can see how the correlation increases in the Gm12878 and K562 cell lines (y-axis) relate to the correlation increases seen in H1-hESCs (x-axis). The increases in all three cell lines are correlated, although the increases in Gm12878 are more modest compared to the other cell lines.

Finally, the plots of Fig. 5f show the comparative trends of the gene expression predicted using the TSS ChIP-seq signals of POL2 (which is directly involved in transcription) and the TSS signal levels of the core predictors. Strikingly, the core predictors are able to predict expression as well as POL2, particularly at low to moderate expression levels.

Taken together, these results suggest that although a small portion of the observed association between TF signal around TSSs and gene expression may be attributable to true TF-binding, for the most part, these associations can be explained by other background/control factors. Particular among those factors is chromatin accessibility, which positively influences both TF/HM signals and expression, thereby explaining most of the correlations between them.

For the sake of completeness, we also predicted gene expression using our core predictors inside whole gene boundaries instead of just around TSSs. We used the number of RNA-seq reads (instead of CAGE reads) to measure gene expression within the gene boundaries. The results obtained were similar, with the contribution of background factors to observed gene expression still being significant, although to a lesser extent compared to when focusing on TSS regions (see Additional file 1: Figure S8).

## Discussion

Using a regression model and a set of 123 ENCODE human ChIP-seq datasets encompassing TFs and HMs across multiple cell lines, we have shown that we can quantify the influences of a variety of other signals on the ChIP-seq signal. The novelty in our treatment lies in the quantitative framework, which shows how genome-wide variations in ChIP-seq signal depend partly on true binding/chromatin state and partly on other, bias-related factors. While we chose to include in our study five such influences that we believe most strongly affect ChIP-seq signals, namely, mappability, GC-content, chromatin accessibility, input DNA control, and IgG control, our framework can readily be extended to quantify the effects of other potential influences. Likewise, the framework can be adapted to use models more sophisticated than linear regression. Regardless of these particular choices, our study emphasizes a mindset in which ChIP-seq datasets are viewed as consisting of an overall genome-wide signal that can be quantifiably decomposed into multiple components. Importantly, we have found that different ChIP-seq datasets can contain substantially different amounts of these components. This suggests that no single control dataset (even cell type-specific iDNA or IgG pulldown) can control equally well for non-specific signal in different ChIP-seq datasets. Rather, each ChIP-seq dataset should be individually analyzed for its specific biases. Our framework also facilitates the separation of the extraneous influences from the binding signal, and even helps to account for correlations between the influences themselves—which can often create false impressions of biological relationships between unrelated datasets.

Among the five non-binding (background) influences we considered, mappability and chromatin accessibility generally exert more influence on the ChIP-seq signal (in terms of predictive power) than the other three. Transcription factor ChIP-seq data demonstrated greater overall predictability using background compared to histone mark data. Higher predictability, of course, means that the total non-binding influences on the signal are greater, and it suggests that a smaller portion of the signal reflects what is truly of biological interest (binding for TFs and modifications for HMs). This may be because most TFs bind DNA in a relatively focal manner, so the true signal is restricted to a small portion of the genome. By contrast, some histone marks can span many thousands or even millions of base pairs, so the total portion of genuine signal is greater in these cases. We also saw that predictability in the Gm12878 and K562 cell lines is greater than that in H1-hESC, and that the influence of the input DNA factor in particular was greater.

It may be worth noting that when predicting background signal levels within the peaks of known ‘pioneer’ TFs [53, 54], the background model may have to be adjusted by weighing down or even excluding the chromatin accessibility feature for better accuracy. This is because the unadjusted chromatin accessibility component (which actually signifies open chromatin) may be an overestimate, considering that pioneer TFs are believed to bind to condensed chromatin leading, in turn, to an open chromatin only thereafter. On the other hand, when pioneer TFs are part of the training set itself, we do not expect any significant impact on our model’s accuracy because the model is fitted over windows tiled across the entire genome. In comparison, the peak regions of a pioneer TF would cover only a minute fraction of the genome. And finally, although some of what is captured by our model may be ‘opportunistic’ binding [54], interestingly, it has been shown that even a ChIP-seq on a factor that has been knocked out results in significant signal levels [55]. Therefore, the ‘background’ signals we remove are not just limited to opportunistic or non-specific binding.

As we expanded the scale of our analysis by increasing the window size, we found that the overall predictability of ChIP-seq data using all influences together steadily improves. The dynamics among the predictive powers of individual influences, however, varies widely, with some increasing in predictive power and others decreasing. These trends can be understood in light of the fact that, just as genomic analyses are conducted at different scales (1 bp resolution for SNP analysis, ~100 bp resolution for TF ChIP-seq peak-calling, and ~100 bp to ~100 kb resolution for chromatin domain analysis), bias effects also occur with varying strengths at different scales [23]. At a given scale, only certain biases are active enough to significantly influence the data. Therefore, an a priori knowledge of the strengths of biases as a function of scale will allow one to prioritize their mitigation. While the reasons for these trends are not obvious, the overall increase in predictability with scale is to be expected. Larger genomic windows represent greater averaging, and therefore variability due to the inherent “noise” of random sampling from a genomic signal tends to even out.

We validated the effectiveness of our model in capturing non-binding influences and separating them from the ChIP-seq signal by showing it leads to a better ranking of peaks and a better correlation between the estimated binding signal and motif frequencies. While the estimated binding signal is of good utility and can be conveniently computed for a given dataset using our software package, by extension, we can envision a complete ChIP-seq analysis methodology where the raw ChIP-seq signal would be subjected to a comprehensive multistage decomposition process, taking into account all relevant control datasets at the same time. Successive decomposition stages would quantify and thereby eliminate the effects of biases one by one, eventually leading to a “purified” ChIP-seq signal that would exclusively reflect binding. In our opinion, such a generalized methodology would more completely address the problems associated with biases in the ChIP-seq technology compared to existing peak-calling methodologies, improving, in the process, the utility of the technology itself.

Finally, we investigated the validity of associations between gene expression and ChIP-seq reads in small windows centered at the TSSs, which have been reported in multiple studies [30, 31]. (We also extended our investigation over whole gene boundaries, see Additional file 1: Figure S8). While the associations may be statistically genuine, our analysis suggests that they are mainly caused by the common factor of chromatin accessibility, which is related to gene expression and also creates a bias towards a higher ChIP-seq signal, regardless of true binding. Several previous studies carried out in yeast [32–34] have already shown this phenomenon to be true, calling it the *expression bias* or *hyper*-*ChIPability*. These essentially refer to the fact that highly transcribed genomic regions are more susceptible to non-specific protein binding compared to other regions. As a result, unrelated transcription factors can bind to these regions, and these effects can sometimes be so strong that transcriptional repressors can erroneously appear to be activators [34]. When such effects can dominate even after selectively filtering for highly enriched peak regions as demonstrated in the yeast studies, it should be no surprise that these effects manifest themselves in TSS regions, particularly when no specific efforts are made to filter the TSSs according to whether they overlap with or are proximal to high-quality peaks. Our results complement those of the yeast studies, and confirm the hyper-ChIPability effects in human using ENCODE datasets. The true cause of these effects may be manifold, such as a very open chromatin resulting from nucleosome depletion or non-specific interactions between antibodies and RNA polymerases [33]. Regardless of the cause, in light of these findings, it becomes all the more crucial to recognize the role of confounding factors and account for their effects while interpreting observed relationships between biological variables. Upon properly accounting for these effects, we can look forward to much more accurate and biologically meaningful insights into the workings of the complex regulatory mechanisms in living organisms.

## Conclusions

In this study, we have developed a novel framework to quantify the roles of different types of biases in influencing the genome-wide ChIP-seq signal using a large compendium of ENCODE datasets. Our model, along with the accompanying software package, has general applicability, yields a better ranking of peaks and a better estimate of the binding signal than competing methods, and has led to several other new insights including (1) background influences are greater at larger scales, (2) mappability and chromatin accessibility significantly influence the ChIP-seq signal, (3) transcription factor ChIP-seq signals have a higher proportion of non-binding influences compared to histone mark ChIP-seq signals, and (4) confounders need to be accounted for before measuring relationships between gene expression and ChIP-seq signals around TSSs. In addition, our study has revealed that due to wide variability in the proportions of biases across different ChIP-seq datasets, no single control dataset can effectively account for all biases, even within similar cellular conditions. Controls that are more specific towards individual biases must therefore be utilized. We expect these observations to significantly advance our understanding of the role of biases in influencing ChIP-seq signals, thereby paving ways for better techniques for bias identification and removal.

## Methods

### ChIP-seq data processing

The 123 human (hg19) ENCODE ChIP-seq datasets (30 TFs and 11 histone marks) corresponding to the three Tier 1 cell lines (Gm12878, H1-hESC, and K562) were downloaded as BAM files from the ENCODE section of the UCSC Genome Browser [56]. For easy access to the read locations, the BAM files were then converted to the BED format using the “bamTobed” utility from the BEDTools package [57]. We removed all duplicate reads. That is, multiple reads mapping to the same position of the same strand of the same chromosome were collapsed into a single representative read. As a result, a genomic window of size $N$ may have a maximum of $2N$ reads in it, one on each strand at each position within the window.

For a selected subset of the TF datasets (17 out of 30, Additional file 5: Table S4) for which a clear DNA-binding motif was available in the JASPAR database [45], peaks were called using MACS v1.4.2 with default parameters. These peak summits were used for the motif correlation analysis in Fig. 4.

For each cell line, the DNaseI hypersensitivity, input DNA control, and the IgG control ChIP-seq datasets were also downloaded and processed as described above.

### Mappability and GC-content data tracks

Our mappability and GC-content tracks are based on the reference human genome version hg19. Across the cell lines, there can be minor differences in these tracks because (1) GM12878 and K562 come from specific individuals and (2) they have mutated genomes. Partly due to lack of availability of genomes specific to the different cell lines and partly because we believe these differences are subtle enough not to significantly affect our results, we ignore them for our investigation. These tracks are thus assumed to be identical for all three cell lines. Both the tracks are binarized in that a given base pair location carries a ‘1’ in the mappability track if it is mappable and a ‘1’ in the GC-content track if it has a G or a C. In the opposite situations, the base pair location carries a ‘0’. A given window of base pairs, thus, will reflect the exact fraction of the total number of base pairs in the window that are mappable or contain a G/C in the mappability and the GC-content tracks, respectively.

### The genome-wide staged regression model

For the staged regression model, the genome was divided into consecutive windows of size (2^7^ + 1) = 129 base pairs. The odd-numbered window sizes were specifically chosen for symmetry around the midpoint. Read counts for the ChIP-seq datasets and bit counts for the mappability and GC-content tracks were then tabulated for the windows. Chromosome Y was excluded from the analysis for the sake of consistency, since some ChIP-seq datasets had reads for chromosome Y while others did not. The regression model was then trained in successive stages by introducing a new predictor at each stage in the order Mappability, GC-content, DNaseI hypersensitivity, input DNA control, and IgG control. If $\varvec{y}_{\varvec{i}}$ denotes the regressand containing the true number of windowed reads, $\varvec{X}_{\varvec{i}}$ the predictor design matrix, and $\varvec{\beta}_{\varvec{i}}$ the coefficient vector for stage $i$, then the ordinary least squares (OLS) estimator is given by $\varvec{\beta}_{\varvec{i}} = \left( {\varvec{X}_{\varvec{i}}^{\varvec{T}} \varvec{X}_{\varvec{i}} } \right)^{ - 1} \varvec{X}_{\varvec{i}}^{\varvec{T}} \varvec{y}_{\varvec{i}}$. The mean squared error (MSE) for stage $i$, denoted $MSE_{i}$, would then be $\left[ {\mathop \sum \limits_{j = 1}^{n} \left( {\varvec{y}_{{\varvec{ij}}} - \varvec{X}_{{\varvec{ij}}}\varvec{\beta}_{{\varvec{ij}}} } \right)^{2} } \right]/(n - 1)$, where $n$ is the number of residuals. The *percentage of variance* (POV) explained by the latest predictor added to the regression in stage  $i$ can then be calculated as $POV_{i} = 100\left( {MSE_{i - 1} - MSE_{i} } \right)/var(\varvec{y}_{\varvec{i}} )$. We use the POV calculated in this manner to measure the predictive power of the individual predictors.

### Motif analysis

To conduct motif analysis for the model evaluation in Fig. 4, we first searched the JASPAR database for binding motifs corresponding to the set of TFs we have in our investigation. Unambiguous DNA-binding motifs were available for only 17 of the 30 TFs we considered. JASPAR count matrix (.cm) files were downloaded for these TFs, which were then converted to position-specific scoring matrix (.pssm) files using the ‘jaspar2meme’ utility from the MEME package [58]. FIMO [46], with default parameters, was then used to identify individual motif locations within peaks. Tasks such as FASTA sequence extraction in peak intervals and counting of the identified motifs within peaks were completed using in-house scripts.

### Gene expression analysis

For the gene expression analysis in Fig. 5, GENCODE v19 gene annotations were downloaded from the UCSC Table Browser along with CAGE expression reads corresponding to the three cell lines. Then, to establish confidence intervals for the predicted expressions, a tenfold cross-validation analysis was carried out as follows. The set of TSSs was randomly divided into ten roughly equal-sized groups. In each fold, the regression model was first trained on nine groups and then used to make expression predictions on the left-out group. The predicted expression values and the Pearson’s correlation coefficient (PCC) between the ground truths and the predictions were recorded.

### Software

The framework described here has been implemented in R and MATLAB, and these packages can be downloaded from http://www.perkinslab.ca/Software.html. The software can be used to first build the background model corresponding to a ChIP-seq dataset, and then estimate the purified binding signal for a user-given set of genomic intervals (e.g., peaks).

## Additional files

**Additional file 1:** This PDF file contains additional plots that were not shown in the main figures
**Additional file 2: Table S1**. This Excel sheet lists the percentages of variance explained by individual predictors for the Gm12878 cell line (relates to main Fig. 2)
**Additional file 3: Table S2**. This Excel sheet lists the percentages of variance explained by individual predictors for the H1-hESC cell line (relates to main Fig. 2)
**Additional file 4: Table S3.** This Excel sheet lists the percentages of variance explained by individual predictors for the K562 cell line (relates to main Fig. 2)
**Additional file 5: Table S4.** This PDF file lists the TFs for which a clear DNA-binding motif was available in the JASPAR database, along with the motif logos
**Additional file 6: Table S5.** This Excel sheet presents a comparative table of Pearson’s correlation coefficients between different binding-signal metrics and DNA-binding motif occurrences under two conditions: (1) when looking at just the motif presence/absence and (2) when looking at the actual motif counts

## Abbreviations

BIDCHIPS: bias decomposition of chip-seq signals
CAGE: cap analysis of gene expression
ChIP-chip: chromatin immunoprecipitation (ChIP) with DNA microarray (chip)
ChIP-seq: chromatin immunoprecipitation and sequencing
ENCODE: encyclopedia of DNA elements
HM: histone marks
iDNA: input DNA
PCC: Pearson’s correlation coefficient
PCR: polymerase chain reaction
POV: percentage of variance
TF: transcription factor
TSS: transcription start site
