# Supplementary material for: BIDCHIPS: bias decomposition and removal from ChIP-seq data clarifies true binding signal and its functional correlates
Source: Epigenetics Chromatin. 2015 Sep 17;8:33. doi: 10.1186/s13072-015-0028-2 (PMC4574076; doi:10.1186/s13072-015-0028-2)
Supplement: Supplementary file 1 — Additional file 1: This PDF file contains additional plots that were not shown in the main figures [file 13072_2015_28_MOESM1_ESM.pdf]

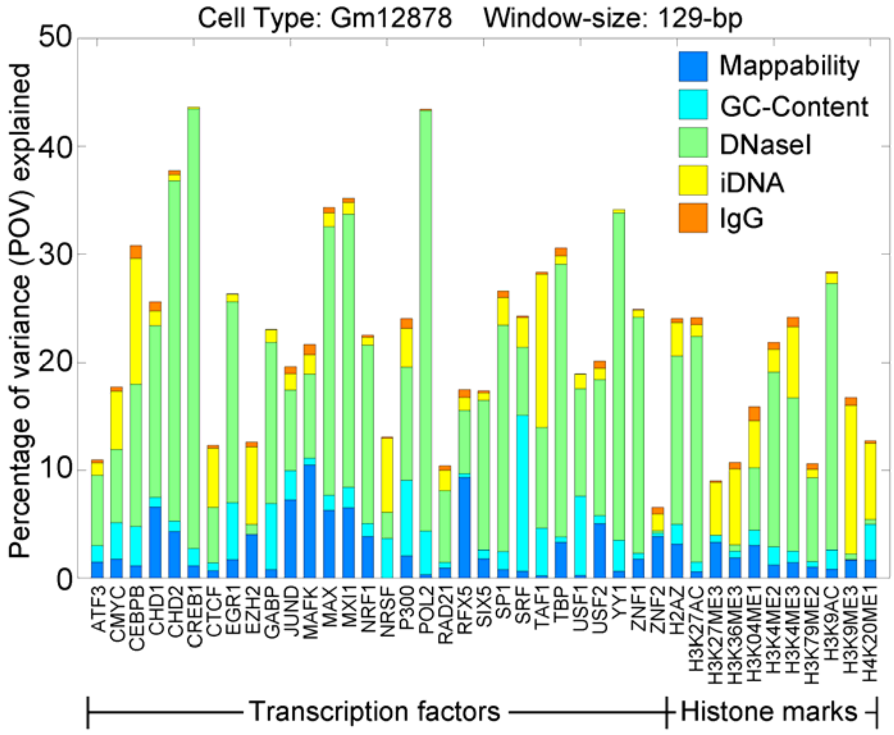

Fig. S1 Stacked bar plot for the Gm12878 cell line showing the predictive power of different predictors in terms of the POV explained.

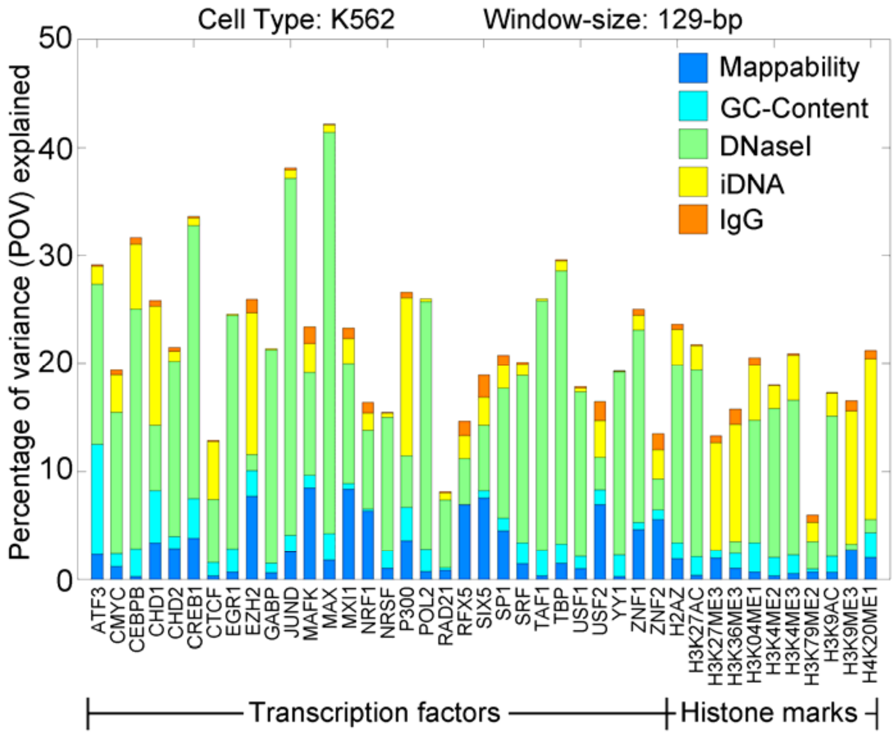

Fig. S2 Stacked bar plot for the K562 cell line showing the predictive power of different predictors in terms of the POV explained.

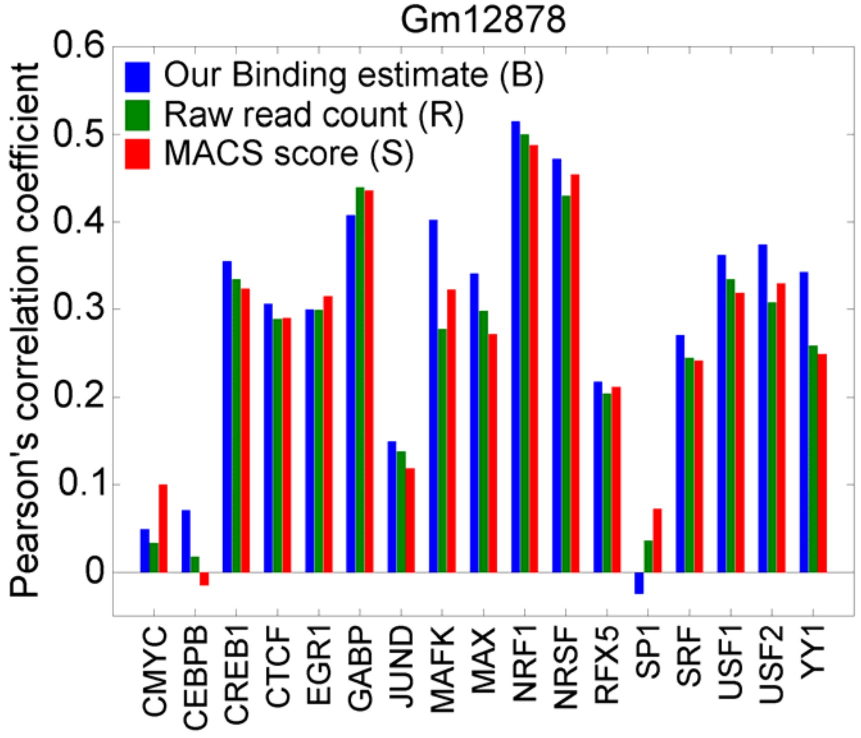

Fig. S3 Pearson's correlation coefficients between different model scores in peaks and DNA-binding motif counts computed for all 17 TFs from the Gm12878 cell line.

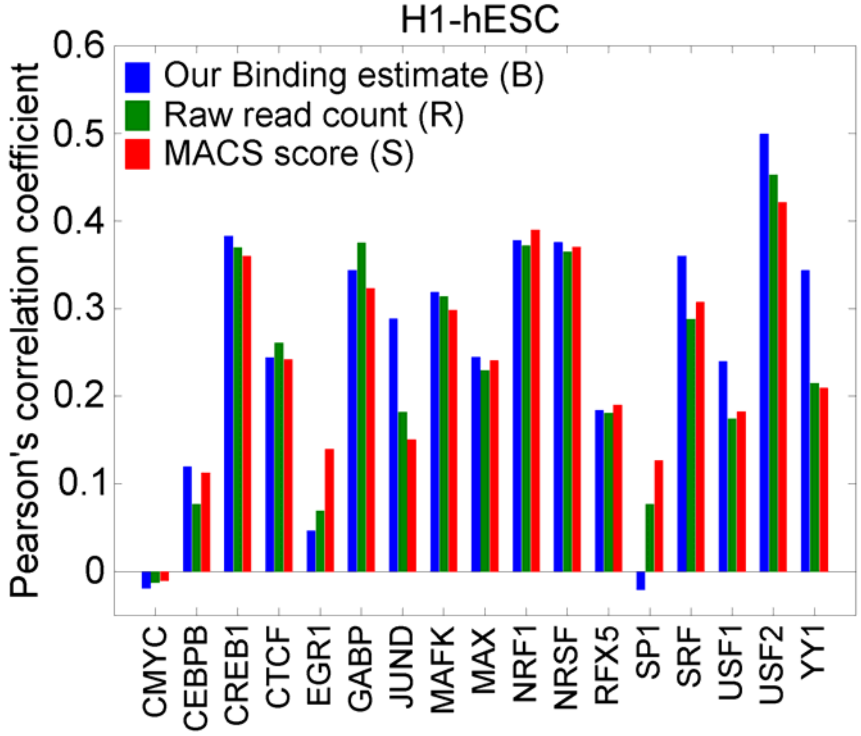

Fig. S4 Pearson's correlation coefficients between different model scores in peaks and DNA-binding motif counts computed for all 17 TFs from the H1-hESC cell line.

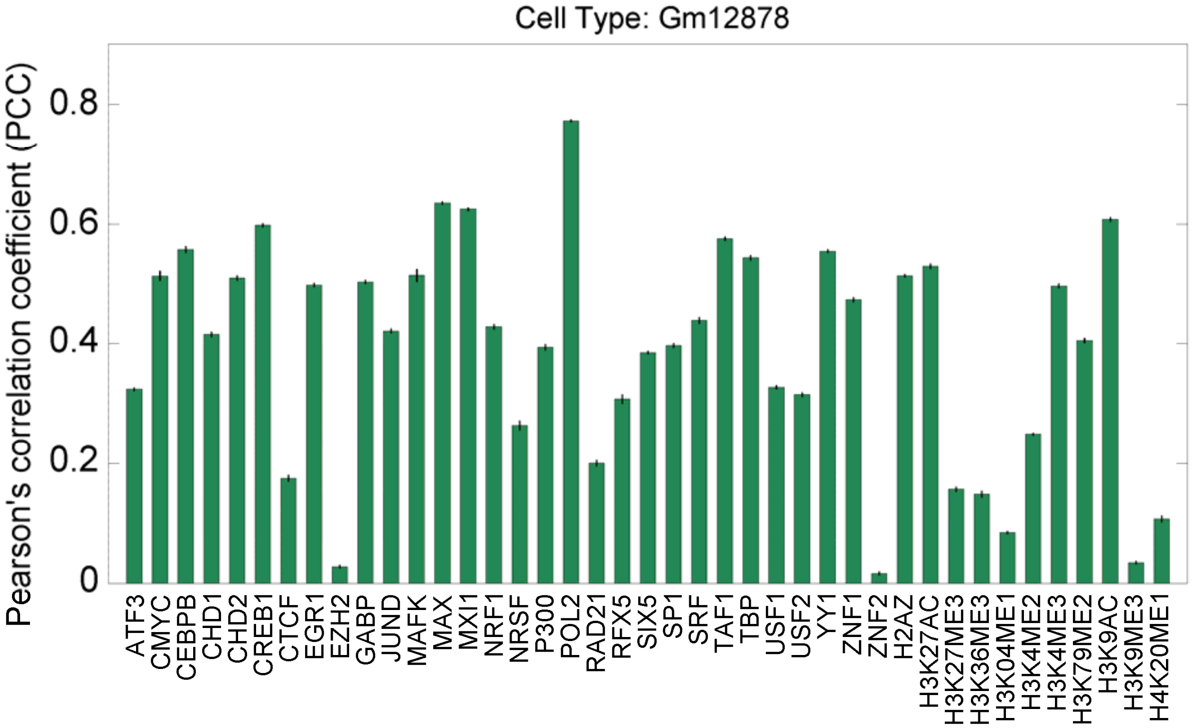

Fig. S5 Pearson's correlation coefficients between true and predicted gene expression, predicted using individual TFs and histone marks for Gm12878.

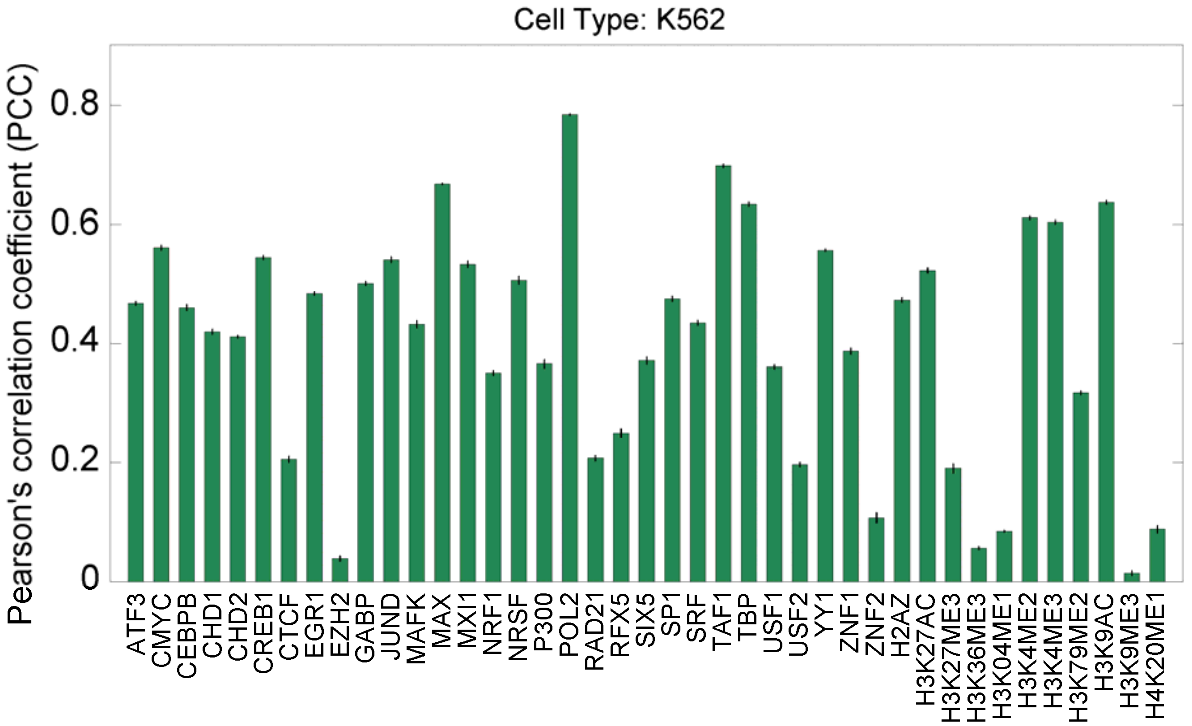

Fig. S6 Pearson's correlation coefficients between true and predicted gene expression, predicted using individual TFs and histone marks for K562.

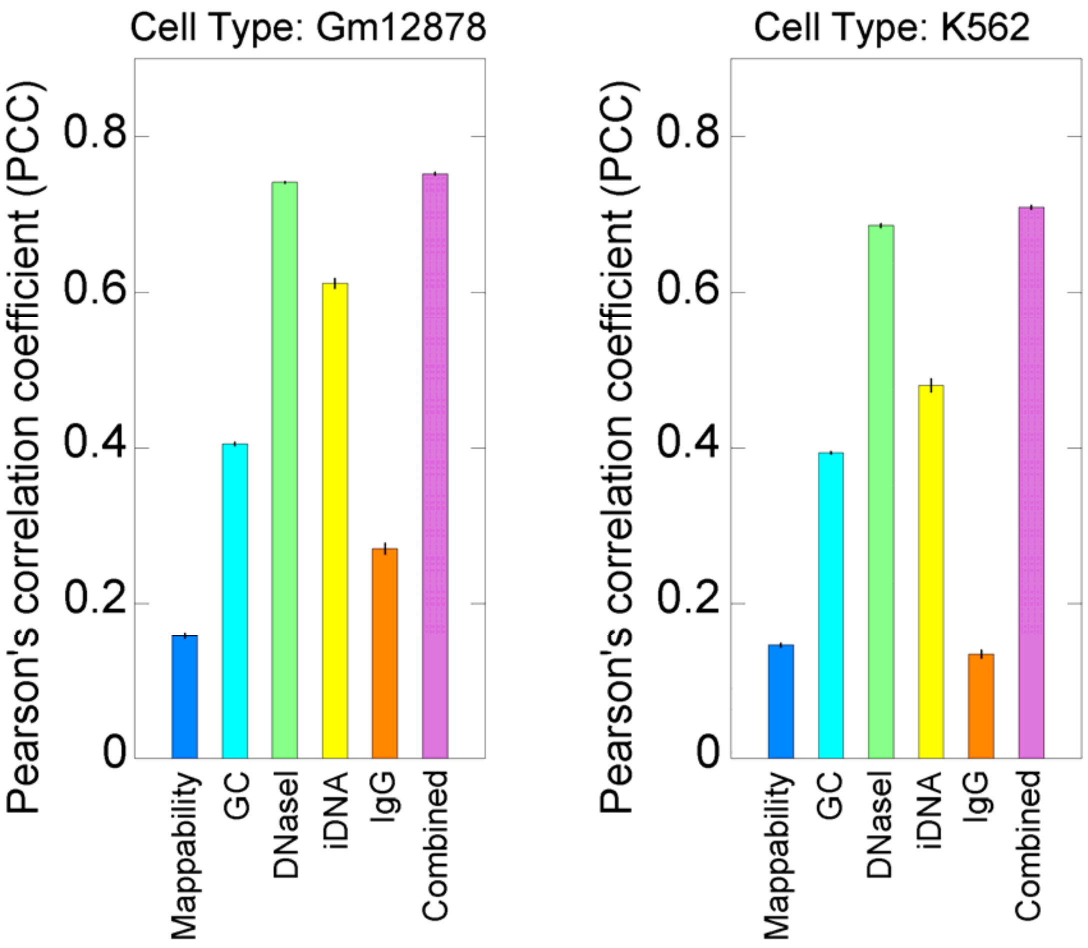

Fig. S7 Pearson's correlation coefficients between true and predicted gene expression (just around TSSs), predicted using core predictors for Gm12878 and K562.

Cont'd on next page …

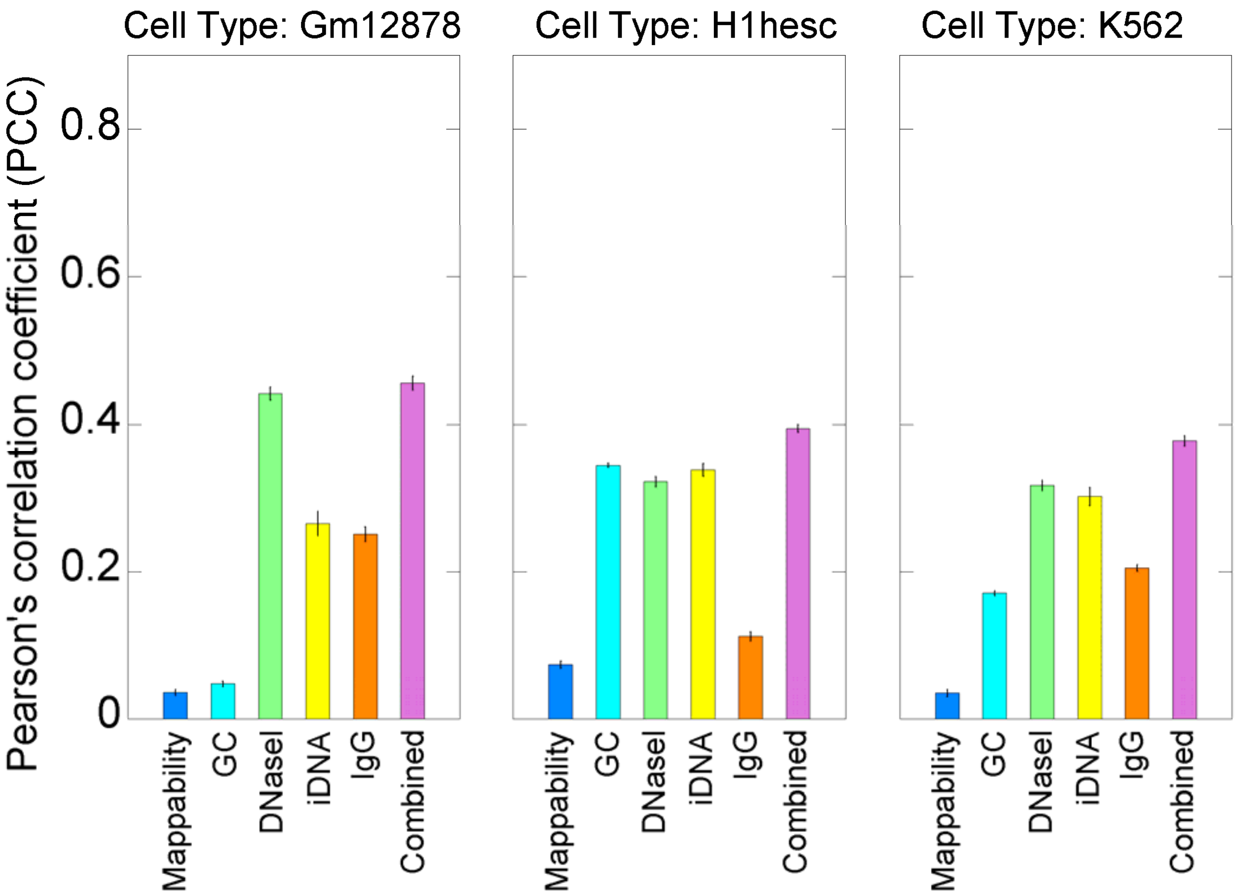

Fig. S8 Pearson's correlation coefficients between true and predicted gene expression for whole-gene boundaries (instead of just around TSSs), predicted using the core predictors. RNA-seq read counts were used to measure expression. These plots reveal that contribution of background factors to observed gene expression is still significant.
