# Supplementary material for: BIDCHIPS: bias decomposition and removal from ChIP-seq data clarifies true binding signal and its functional correlates
Source: Epigenetics Chromatin. 2015 Sep 17;8:33. doi: 10.1186/s13072-015-0028-2 (PMC4574076; doi:10.1186/s13072-015-0028-2)
Supplement: Supplementary file 5 — Additional file 5: Table S4. This PDF file lists the TFs for which a clear DNA-binding motif was available in the JASPAR database, along with the motif logos [file 13072_2015_28_MOESM5_ESM.pdf]

TFs and MOTIFs

Below is the list of TFs for which a clear DNA-binding motif was available in the JASPAR database, along with the motif logos.

TF Name	Sequence Motif Logo
CMYC	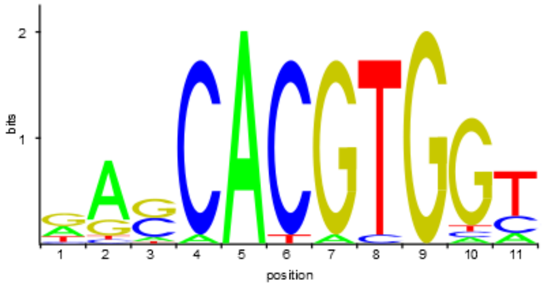
CEBPB	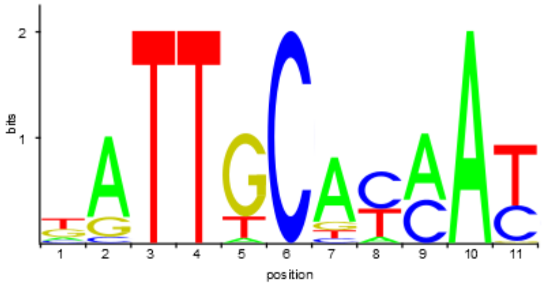
CREB1	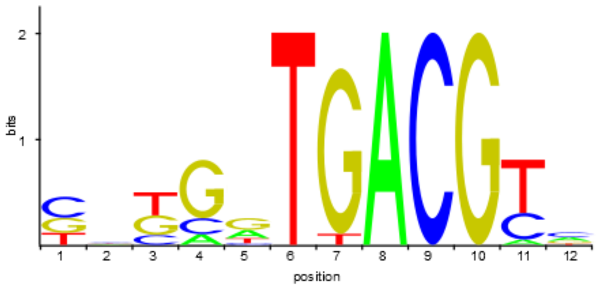

CTCF	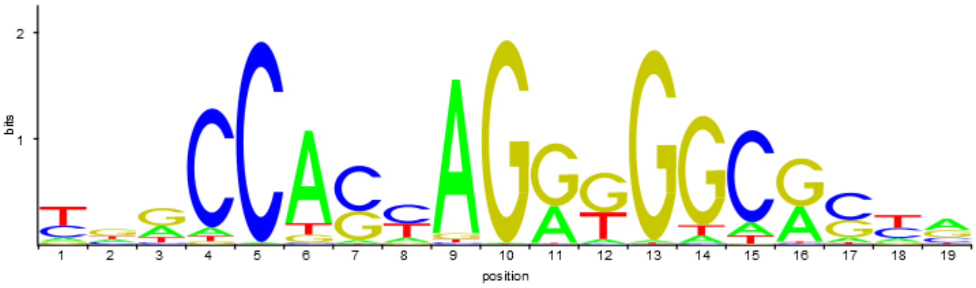
EGR1	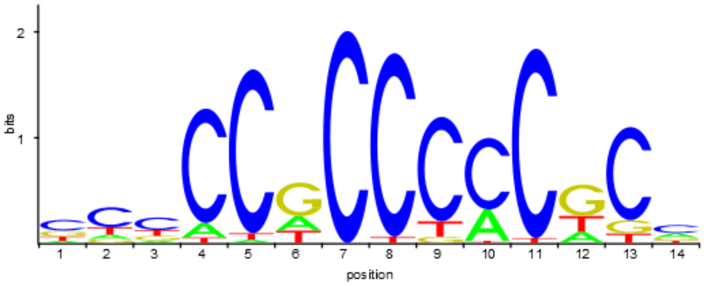
GABP	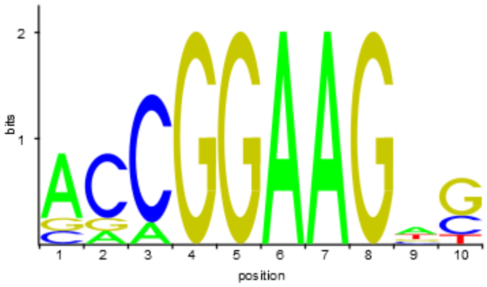
JUND	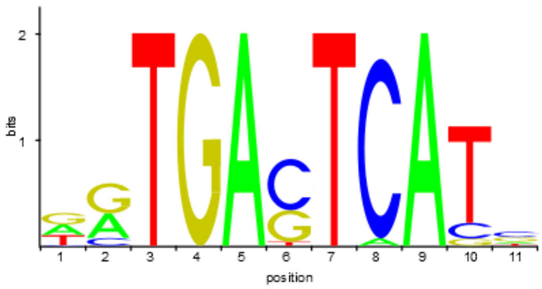

MAFK	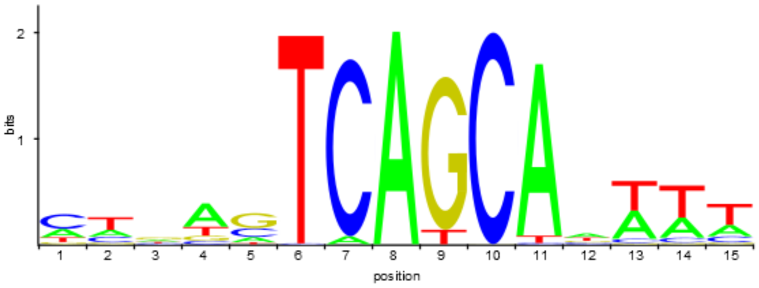
MAX	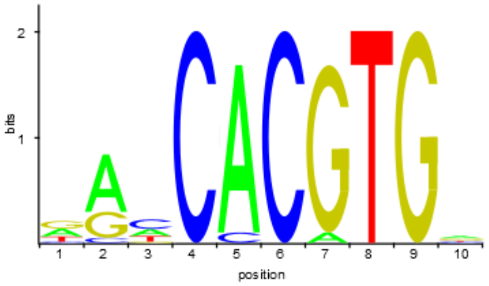
NRF1	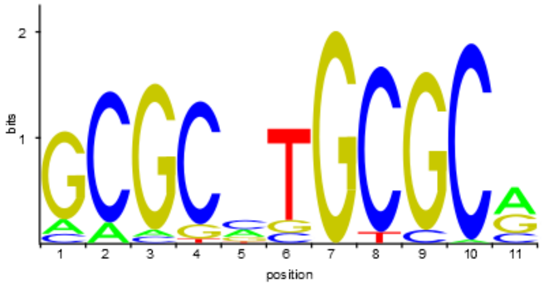
NRSF	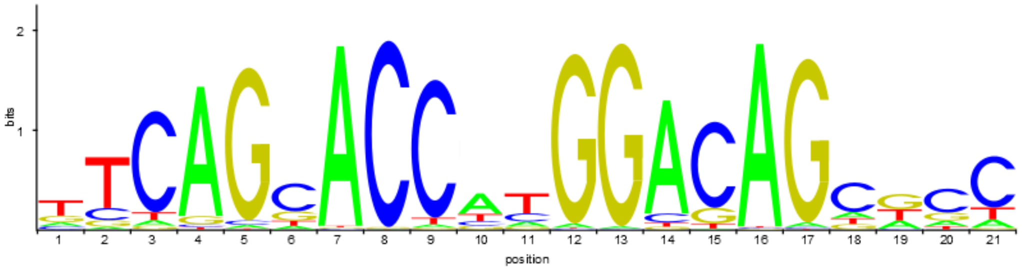

RFX5	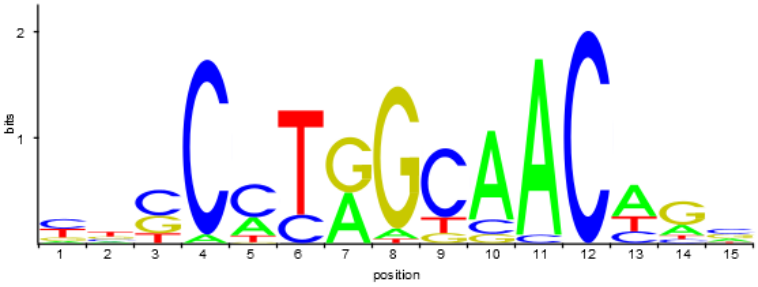
SP1	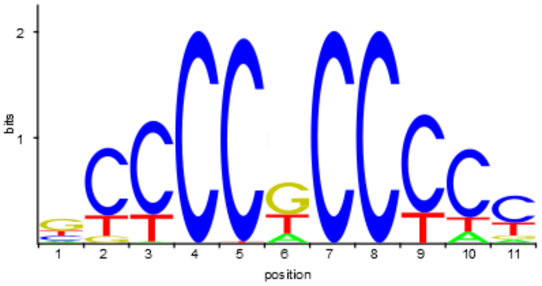
SRF	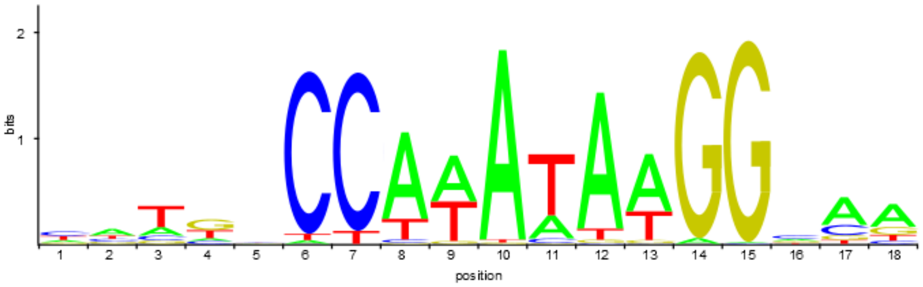
USF1	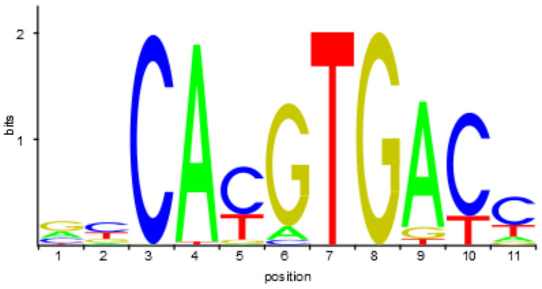

USF2	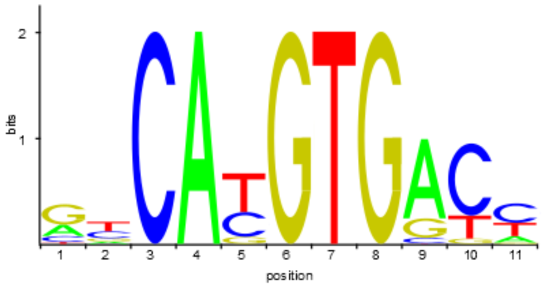
YY1	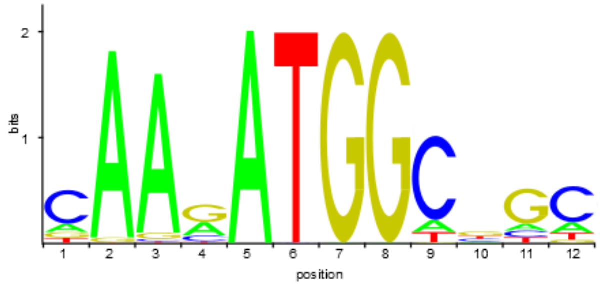
